# Crocin's role in modulating MMP2/TIMP1 and mitigating hypoxia-induced pulmonary hypertension in mice

**DOI:** 10.1038/s41598-024-62900-8

**Published:** 2024-06-03

**Authors:** Jing Deng, Rui-Qi Wei, Wen-Mei Zhang, Chang-Yu Shi, Rui Yang, Ming Jin, Chunmei Piao

**Affiliations:** 1 Beijing Institute of Heart Lung and Blood Vessel Diseases, Beijing Anzhen Hospital Affiliated to the Capital Medical University, Beijing, 100029 China; 2 Department of Pulmonary and Critical Care Medicine, Beijing Anzhen Hospital Affiliated to the Capital Medical University, Beijing, 100029 China; 3 School of Basic Medical Sciences, Yanbian University, Yanji, 133000 China; 4 Department of Pulmonary and Critical Care Medicine, Beijing Chaoyang Hospital Affiliated to the Capital Medical University, Beijing, 100020 China

**Keywords:** Pulmonary hypertension, Transcriptome sequencing, Matrix metalloproteinase, Matrix metalloproteinase tissue inhibitor, Pulmonary arterial fibroblast, Crocin, Cardiovascular biology, Molecular medicine

## Abstract

To explore the molecular pathogenesis of pulmonary arterial hypertension (PAH) and identify potential therapeutic targets, we performed transcriptome sequencing of lung tissue from mice with hypoxia-induced pulmonary hypertension. Our Gene Ontology analysis revealed that “extracellular matrix organization” ranked high in the biological process category, and matrix metallopeptidases (MMPs) and other proteases also played important roles in it. Moreover, compared with those in the normoxia group, we confirmed that *MMPs* expression was upregulated in the hypoxia group, while the hub gene *Timp1* was downregulated. Crocin, a natural MMP inhibitor, was found to reduce inflammation, decrease MMPs levels, increase *Timp1* expression levels, and attenuate hypoxia-induced pulmonary hypertension in mice. In addition, analysis of the cell distribution of MMPs and *Timp1* in the human lung cell atlas using single-cell RNAseq datasets revealed that *MMPs* and *Timp1* are mainly expressed in a population of fibroblasts. Moreover, in vitro experiments revealed that crocin significantly inhibited myofibroblast proliferation, migration, and extracellular matrix deposition. Furthermore, we demonstrated that crocin inhibited TGF-β1-induced fibroblast activation and regulated the pulmonary arterial fibroblast MMP2/TIMP1 balance by inhibiting the TGF-β1/Smad3 signaling pathway. In summary, our results indicate that crocin attenuates hypoxia-induced pulmonary hypertension in mice by inhibiting TGF-β1-induced myofibroblast activation.

## Introduction

Pulmonary hypertension is a vascular disease associated with severe morbidity and mortality^1^ that is characterized by pulmonary vascular remodeling and extracellular matrix deposition^2^. Currently, targeted drugs aimed at relieving pulmonary vasoconstriction have widely used in pulmonary hypertension patients. However, these drugs fail to alleviate pulmonary vascular remodeling and restore right ventricular function, and lung transplantation is ultimately the only curative option^3,4^. Therefore, identifying the key cells and pathways in pulmonary vascular remodeling may lead to the discovery of new therapeutic targets for pulmonary hypertension.

Recently, studies have used transcriptome sequencing and bioinformatics analysis to screen differentially expressed genes (DEGs) in pulmonary hypertension and further investigate potential biomarkers and regulatory targets. Several transcriptome studies have analyzed the DEGs in cells from patients with idiopathic PH and in the lung tissue of rat models of pulmonary hypertension induced by monocrotaline; these studies have found that DEGs are mainly enriched in the abnormal proliferation of smooth muscle cells and endothelial cells, as well as in inflammatory reactions^5–10^. Another transcriptome study compared 32 mouse strains exposed to chronic hypoxia and found that right ventricular systolic pressure (RVSP) was significantly elevated in the PL/J mouse strain, as well as that the immune inflammatory reaction was the most noticeably enriched^11^. Although these transcriptome sequencing studies on both humans and experimental animals have shown promising results, further research is needed to better understand the mechanisms of pulmonary hypertension disease models and to find effective therapeutic targets for pulmonary vascular remodeling, which involves not only the proliferation and phenotypic transformation of pulmonary artery vascular cells, but also the complex interaction between outer pulmonary artery fibroblasts (PAFs) and the extracellular matrix (ECM). As an important component of the adventitia, the activation, migration, and excessive proliferation of fibroblasts may represent important changes in the early stage of pulmonary vascular remodeling^12,13^; however, these processes have received little attention. Fibroblasts can be activated to become myofibroblasts when tissue is damaged by hypoxia, which can also lead to the deposition of ECM proteins, such as collagen, fibronectin, tendinin, and elastin^14,15^. Hypoxia upregulates the expression of hypoxia-inducible factor-1α (HIF-1α), vascular endothelial growth factor-A (VEGF-A), and matrix metalloproteinases (MMPs) in myofibroblasts, thus promoting myofibroblast proliferation and migration to the inner membrane. These changes disrupt the balance of tissue inhibitors of proteolytic enzymes (TIMPs) and ECM enzymes (such as MMPs, elastase, and lysyl oxidase) in PH, followed by ECM deposition and increased elastin breakdown in the pulmonary arteries, which leads to pulmonary arterial remodeling^1^. Therefore, targeting the dynamic balance of the ECM to reduce ECM deposition can be a novel therapeutic option for treating pulmonary hypertension.

Crocin, a carotenoid component^16,17^, has numerous benefits, such as antiatherosclerosis, antioxidant, free radical clearance, anti-inflammatory, and anticancer effects^18–21^. At present, there have been only a few studies on the pharmacological effects of crocin in rats; for instance, crocin exhibits hypotensive effects in rats^22,23^, while also attenuating pulmonary inflammation and pulmonary vascular dysfunction in a rat model of bleomycin-induced pulmonary fibrosis^24^. Crocin can also inhibit inflammation or oxidative stress in monocrotaline-induced pulmonary hypertension in rats^25,26^. Based on these studies, crocin has been found to alleviate inflammation in rat disease models, thereby playing a protective role in the disease progression. In contrast, some studies have demonstrated that crocin regulates MMPs; for example, it can reduce the gene expression of *MMPs* in MCF-7 breast cancer cells when treated with crocin water extract^27^. Moreover, crocin can also significantly reduce atherosclerosis by inhibiting MMP 2 and MMP 9, which are produced by inflammatory cells^28^. Qi et al. also found crocin to be a natural inhibitor of matrix metalloproteinases, which can alleviate the formation of aortic aneurysm and dissection in mice^29^. Given that the above studies demonstrated the inhibitory effect of MMPs, we hypothesized that crocin might ameliorate PAH by regulating the dynamic balance of the ECM. In the present study, we used transcriptome sequencing to verify the increase in ECM deposition and the decrease in the expression of the hub gene TIMP1 in mice with hypoxia-induced pulmonary hypertension (HPH). In addition, we found that crocin can significantly inhibit the occurrence of PAH, reduce ECM deposition, and disrupt the balance between MMPs and TIMP1. Moreover, single-cell RNA sequencing confirmed that *MMP2* and *Timp1* were expressed mainly in adventitial fibroblasts. Furthermore, in vitro experiments verified that crocin regulates the balance between MMP2 and TIMP1 through TGF- β signaling, in addition to ECM changes. Considering the above results, we propose that targeting pulmonary arterial fibroblast activation and ECM may be potential therapeutic strategies for treating pulmonary hypertension.

## Results

### Transcriptome sequencing reveals that MMPs and TIMP1 homeostasis play an important role in hypoxia-induced pulmonary hypertension in mice

**Figure 1:**
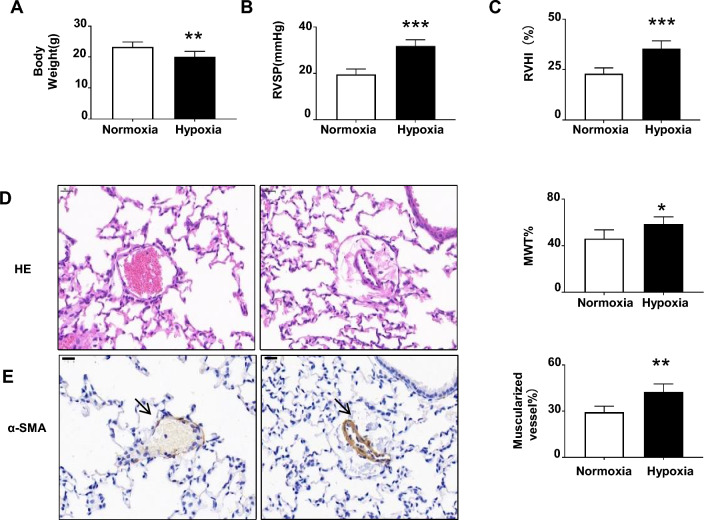
Generation of mice with hypoxia-induced pulmonary hypertension (**A**) Changes in right ventricular systolic pressure (RVSP, mmHg) in the normoxia (n = 8) and hypoxia (n = 7) groups; ***P < 0.001. (**B**) Changes in the right ventricle hypotrophy index (RVHI, %) compared between the normoxia and hypoxia groups, ***P < 0.001. (**C**) Hematoxylin–eosin (HE) staining was used to determine changes in the medial wall thickness (MWT%) between the normoxia and hypoxia groups; *P < 0.05. (**D**) α-smooth muscle actin (α-SMA) staining (arrows) and calculation of muscularized vessels were performed and compared between the normoxia and hypoxia groups; **P < 0.01.

**Figure 2:**
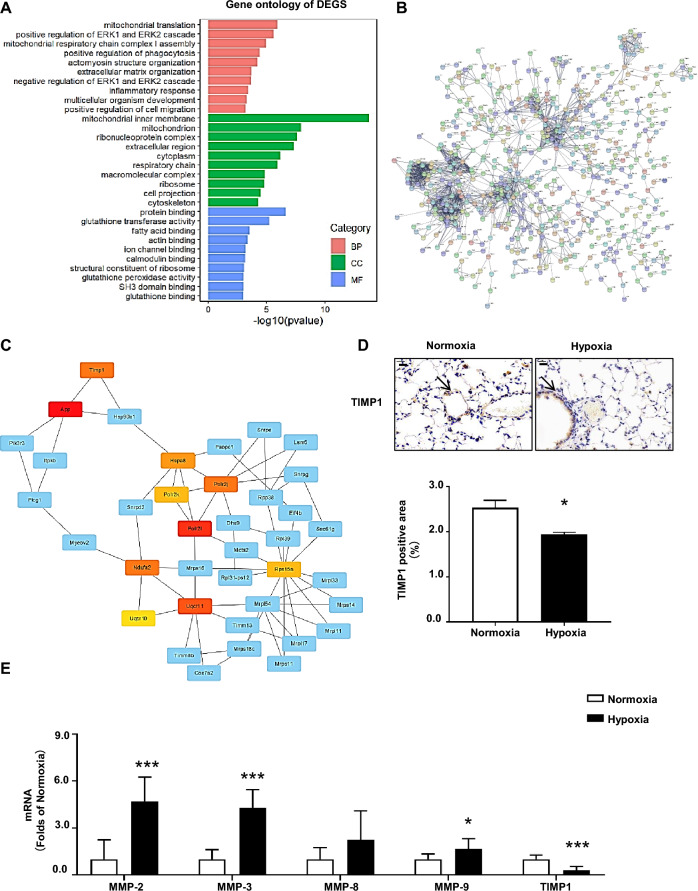
Transcriptomic analysis of lung tissue from mice with hypoxia-induced pulmonary hypertension. (**A**) GO functional enrichment analysis of DEGs between the normoxia and hypoxia groups. (**B**) A differentially expressed gene-protein interaction network based on STRING. Each node in the figure represents a gene or its encoded protein, and the lines represent their interactions. (**C**) Analysis of gene‒protein interactions and hub genes. A PPI subnetwork was constructed with Cytoscape software, and hub genes were screened with the CytoHubba plugin. Each square represents a gene, the line represents the interaction between genes, and red, orange, and yellow represent hub genes. (**D**) The expression levels of TIMP1 in normoxic and hypoxic mouse lung tissue were determined via immunohistochemistry (arrows); scale bars, 20 μm; compared between the normoxic and hypoxic groups, **P < 0.01. (**E**) The expression levels of *MMP-2*, *MMP-3*, *MMP-8*, *MMP-9*, and *Timp1* in normoxic and hypoxic mouse lung tissue were determined via real-time PCR; between the normoxic and hypoxic groups, *P < 0.01, ***P < 0.001.

We established HPH in C57BL/6 mice, and after 4 weeks, the body weight, right ventricular systolic pressure (RVSP), and right ventricular hypertrophy index (RVHI) were checked to confirm pulmonary arterial hypertension (Fig. 1a–c). HE staining and α-SMA staining revealed thickening of the medial wall and muscularization of the pulmonary arteriole (Fig. 1d, e). Transcriptome sequencing of lung tissue from hypoxic and normoxic mice was performed to explore the key signaling pathways involved in PAH. We obtained a total of 1,053 differentially expressed genes (DEGs) in the HPH and normoxic groups, including 455 upregulated and 598 downregulated genes (Supplementary Figure 1). The GO enrichment assay revealed that the biological processes (BPs), cellular components (CCs), and molecular functions (MFs) were mainly related to mitochondrial translation, the ERK1 and ERK2 cascades, phagocytosis, actomyosin structure organization, and extracellular matrix organization (Fig. 2a). KEGG pathway enrichment analyses revealed that the DEGs were associated mainly with oxidative stress, smooth muscle cell proliferation, and inflammatory response (Supplementary Figure 1). In addition, we explored the interactions between proteins corresponding to the DEGs via an online analysis based on the Search Tool for the Retrieval of INteracting Genes/proteins (STRING) database and constructed a PPI network (Fig. 2b). The PPI network data constructed using STRING were imported into Cytoscape software, and the CytoHubba plug-in was used to identify the hub genes that play key roles in the process of pulmonary hypertension (Table 1, Fig. 2c). Among the 10 hub genes, *Timp1*, an MMP inhibitor, was found to be involved in pulmonary hypertension. We verified that *Timp1* was downregulated in the lung tissues of HPH mice through transcriptome sequencing, which was consistent with the immunohistochemistry results (Fig. 2d). Quantitative real-time PCR (qRT‒PCR) showed that, compared with those in the normoxia group,* MMP-2*, *MMP-3*, and *MMP-9* expression was increased in the hypoxia group, while TIMP1 expression was reduced in the hypoxic group (Fig. 1e). Taken together, our results suggest that the MMPs/TIMP1 imbalance in hypoxic pulmonary hypertension may also play an important role in pulmonary hypertension development.

**Table 1:** Top 10 hub genes.

Hub gene	Gene full name	Degree
App	Amyloid beta (A4) precursor protein	68
Polr2l	Polymerase (RNA) II (DNA directed) polypeptide L	60
Uqcr11	Ubiquinol-cytochrome c reductase, complex III, subunit XI	56
TIMP1	Tissue inhibitor of metalloproteinase 1	52
Ndufa2	NADH ubiquinone oxidoreductase subunit A2	52
Polr2j	Polymerase (RNA) II (DNA directed) polypeptide J	52
Hspa8	Heat shock protein 8	50
Polr2k	Polymerase (RNA) II (DNA directed) polypeptide K	48
Rps15a	Ribosomal protein S15A	48
Uqcr10	Ubiquinol-cytochrome c reductase, complex III, subunit X	44

### Crocin inhibits vascular remodeling and inflammation in hypoxia-induced pulmonary hypertension

**Figure 3:**
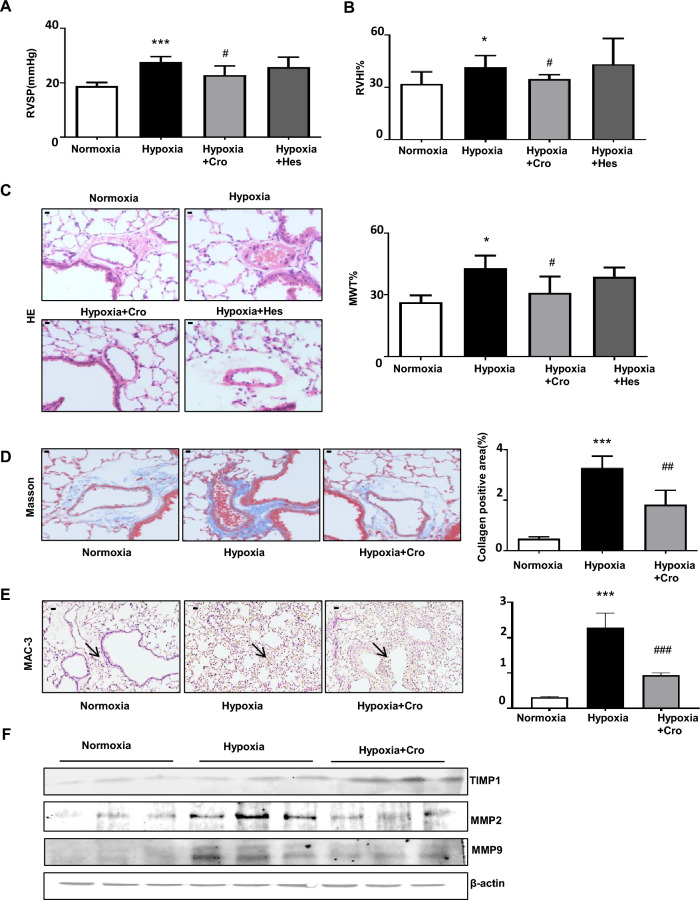
Crocin inhibits pulmonary vascular remodeling in mice with hypoxia-induced pulmonary hypertension. (**A**) Changes in right ventricular systolic pressure (RVSP, mmHg); n = 6 for each group; crocin (50 mg/kg) or hesperetin (50 mg/kg) was administered; ***P < 0.001 compared with the normoxia group; ^#^*P* < 0.05 compared with the hypoxia group. (**B**) Changes in the right ventricle hypotrophy index (RVHI, %) compared with normoxia, *P < 0.05; compared with the hypoxia group, ^#^*P* < 0.05. (**C**) Hematoxylin–eosin (HE) staining, changes in medial wall thickness% (MWT%), compared with normoxia group, *P < 0.05; compared with hypoxia group, ^#^*P* < 0.05. (**D**) Masson staining and quantification of collagen deposits. Compared with the normoxia group, ***P < 0.001; compared with the hypoxia group, ^##^*P* < 0.01; scale bars, 50 μm. (**E**) MAC-3 staining (arrows) and quantification of MAC-2 positive cells. Compared with the normoxic group, ***P < 0.001; compared with the hypoxic group, ^###^*P* < 0.001; scale bars, 50 μm. (**F**) The effects of crocin on MMPs and TIMP1 expression in mice with hypoxia-induced pulmonary hypertension. Three mice from each group were compared.

Crocin attenuates pulmonary inflammation and oxidative stress in a rat model of monocrotaline-induced pulmonary arterial hypertension^24–26^; however, the detailed mechanism through which crocin affects pulmonary hypertension has not been determined. To explore the protective effects of natural compounds on mouse HPH, we intraperitoneally administered crocin and hesperetin (50 mg/kg) every three days and found that crocin significantly reduced RVSP and RVHI in HPH mice, while hesperetin had no effect on these parameters (Fig. 3a,b). Hematoxylin and eosin (HE) and Masson staining revealed that crocin significantly reduced pulmonary vascular remodeling in mice with HPH (Fig. 3c,d). We subsequently evaluated inflammation in the lung tissue and found that crocin significantly reduced macrophage infiltration in HPH mice (Fig. 3e). Our transcriptome study (Fig. 1e) revealed a possible imbalance of MMPs/TIMP1 in the extracellular matrix in HPH mice. Qi et al. demonstrated that crocin and hesperetin are MMP inhibitors^29^. We evaluated the activity of MMPs in the murine colorectal cancer SL4 cell line to examine the effect of MMPs on the balance between MMPs and TIMP1. Crocin significantly inhibited MMP2 activity, decreased MMP2 protein levels, and increased TIMP1 levels (Supplementary Figure 2). WB analysis revealed that crocin modulated the lung MMP2/TIMP1 balance (Fig. 3e). Taken together, these findings suggested that crocin modulates vascular remodeling and the MMP2/TIMP1 balance in HPH mice.

### Crocin inhibits the proliferation and migration of pulmonary artery fibroblasts

**Figure 4:**
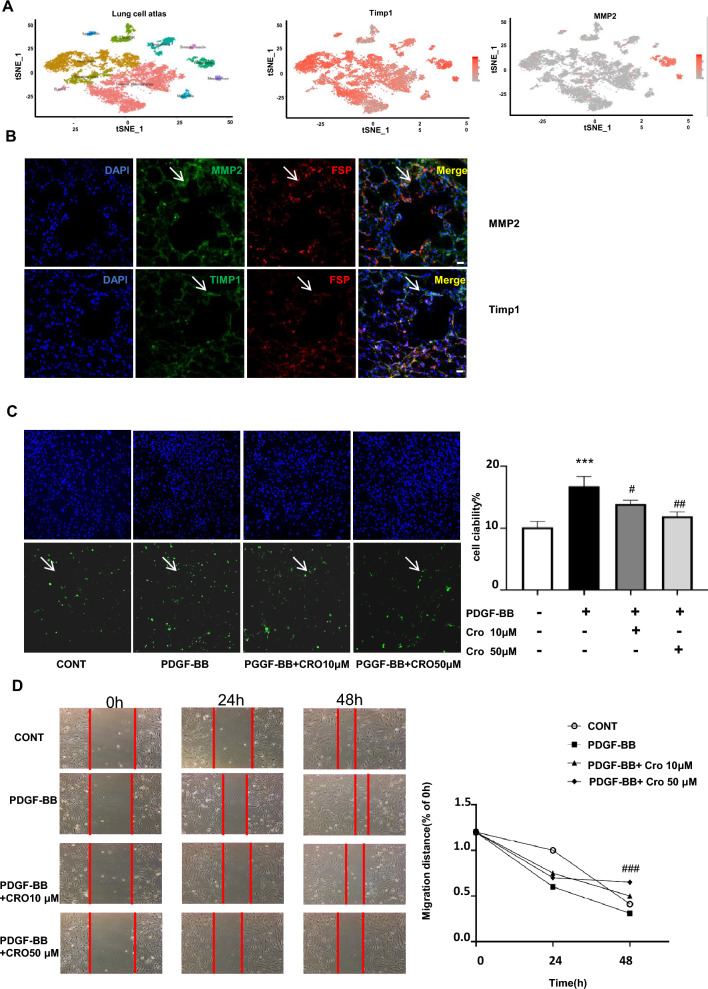
Crocin inhibits the proliferation and migration of pulmonary artery fibroblasts. (**A**) The distribution of *MMP-2* and *Timp1* in the human lung cell atlas of the Sanger Lung scRNA-Seq Database (https://asthma.cellgeni.sanger.ac.uk/). (**B**) Immunofluorescence staining (arrows) of MMP2 (green) or TIMP1 (green) with FSP (red) in lung tissue from mice with hypoxia-induced pulmonary hypertension for 28 days; scale bar, 50 μm. (**C**) The effects of crocin on proliferation assessed by BrdU assay; crocin 10 or 50 µM was applied, followed by stimulation with PDGF-BB (20 ng/ml), compared with the control group, ***P < 0.001; compared with PDGF-BB treated group, ^#^*P* < 0.05, ^##^*P* < 0.01. (**D**) The effects of crocin on migration determined by a scratch wound healing assay were compared with those of the PDGF-BB-treated group; ^###^*P* < 0.001.

Because crocin modulates hypoxia-induced pulmonary vascular remodeling by altering the homeostasis of MMP2 and TIMP1, we assessed the distribution of *MMP2* and *Timp1* in the human lung cell atlas using the Sanger Lung scRNA-Seq Database (https://asthma.cellgeni.sanger.ac.uk/). *MMP-2* was strongly expressed in fibroblasts, while *Timp1* was expressed in fibroblasts, smooth muscle cells, and inflammatory cells, and *MMP-9* was expressed in inflammatory cells in human lung tissue (Fig. 4a). In the HPH mice, immunofluorescence staining demonstrated progressively increased MMP-2 and decreased TIMP1 lung staining that colocalized with FSP (a fibroblast marker) in pulmonary vessels under hypoxia (Fig. 4b). It has also been previously reported that early substantial proliferation and maintenance of an abnormal or persistently activated cellular phenotype, including a proinflammatory phenotype, excessive proliferation, and antiapoptotic effects, were observed in pulmonary vascular cells cultured from PAH patients and animal models in vitro^30^. To explore whether crocin influences fibroblast proliferation and migration, we added PDGF-BB to induce fibroblast activation. A BrdU assay was used to evaluate proliferation, and the results revealed that the number of BrdU-positive cells in the crocin-treated group was significantly lower than that in the hypoxia group, indicating that crocin inhibited fibroblast proliferation (Fig. 4c). In addition, our results showed that the difference in the cell scratch density in each group was not obvious at 24 h. After 48 h, the crocin concentration gradient inhibited the migration of fibroblasts (Fig. 4d). Taken together, these experimental results suggested that crocin inhibits the proliferation and migration of fibroblasts, indicating that crocin participates in fibroblast activation and thereby inhibits vascular remodeling.

### Crocin inhibits pulmonary artery fibroblast activation and regulates the MMP-2/TIMP1 balance

**Figure 5:**
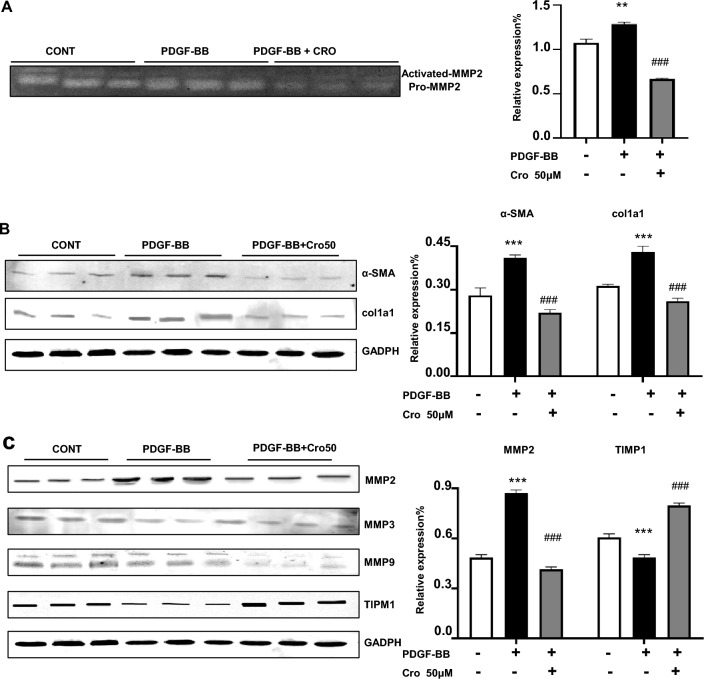
Crocin regulates pulmonary arterial fibroblast MMPs/TIMP1 homeostasis in mice with hypoxia-induced pulmonary hypertension. (**A**) Effect of crocin on MMP-2 activity in pulmonary arterial fibroblasts activated by PDGF-BB (20 ng/ml) or administered crocin (50 mg/kg) compared with that in the normoxia group; **P < 0.01; compared with the hypoxia group, ^###^*P* < 0.001. (**B**) The effects of crocin on pulmonary arterial fibroblast activation induced by PDGF-BB (20 ng/ml) and crocin (50 μM) were compared with those of the control group; ***P < 0.001; compared with the hypoxia group, ^###^*P* < 0.001. (**C**) The effects of crocin on MMP and TIMP1 homeostasis in pulmonary arterial fibroblast activation induced by PDGF-BB (20 ng/ml) and crocin (50 μM) compared with those in the control group; ***P < 0.001; compared with the hypoxia group, ^###^*P* < 0.01.

To explore whether crocin affects MMPs/TIMP1 production by activated fibroblasts, we performed gelatin zymography on pulmonary arterial fibroblasts activated by PDGF-BB and found that MMP2 activity in the cell supernatant was significantly reduced after the administration of crocin (Fig. 5a). We confirmed pulmonary arterial fibroblast transformation by WB, which revealed that the expression levels of proteins associated with myofibroblasts (a-SMA and Col1a1) were decreased (Fig. 5b). Crocin modulates MMPs/TIMP1 homeostasis during pulmonary arterial fibroblast transformation to myofibroblasts after the administration of PDGF-BB (Fig. 5c). Taken together, these findings suggest that crocin inhibits pulmonary arterial fibroblast transformation to myofibroblasts by regulating MMP-2/TIMP1 homeostasis.

### Crocin participates in pulmonary artery fibroblast activation through the TGF-β signaling pathway

**Figure 6:**
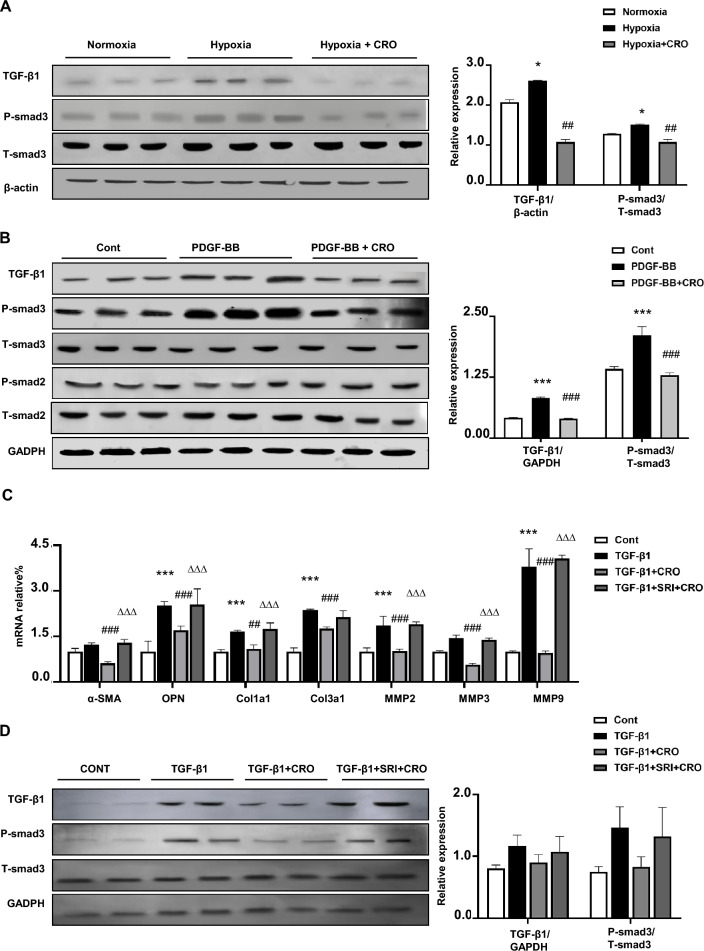
Crocin arrogated pulmonary arterial fibroblast MMP2/TIMP1 via TGF-β1/Smad3 signaling in mice with hypoxia-induced pulmonary hypertension. (**A**) The effects of crocin on TGF-β1/Smad3 signaling in hypoxia-induced pulmonary hypertension in mice administered crocin (50 mg/kg) compared with those in the normoxia group; *P < 0.05; compared with those in the hypoxia group, ^##^*P* < 0.01. (**B**) The effects of crocin on TGF-β1/Smad3 signaling in pulmonary arterial fibroblasts activated by PDGF-BB (20 ng/ml) and administered crocin (50 μM) compared with those in the control group; ***P < 0.001; compared with the hypoxia group, ^###^*P* < 0.001. (**C**) The effects of crocin on pulmonary arterial fibroblast activation by TGF-β1(5 ng/ml) and administered crocin (50 μM) compared with the control group, *P < 0.05, ***P < 0.001; compared with TGF-β1 group, ^##^*P* < 0.01, ^###^*P* < 0.001; TGF-β1 + CRO group, ∆∆∆ P < 0.001. (**D**) The effects of crocin on TGF-β1/Smad3 signaling in pulmonary arterial fibroblasts activated by TGF-β1 (5 ng/ml), administrated crocin (50 μM), and SRI-011381 (10 μM) compared with the control group, **P < 0.01; compared with those in the hypoxia group, ^#^*P* < 0.05.

**Figure 7:**
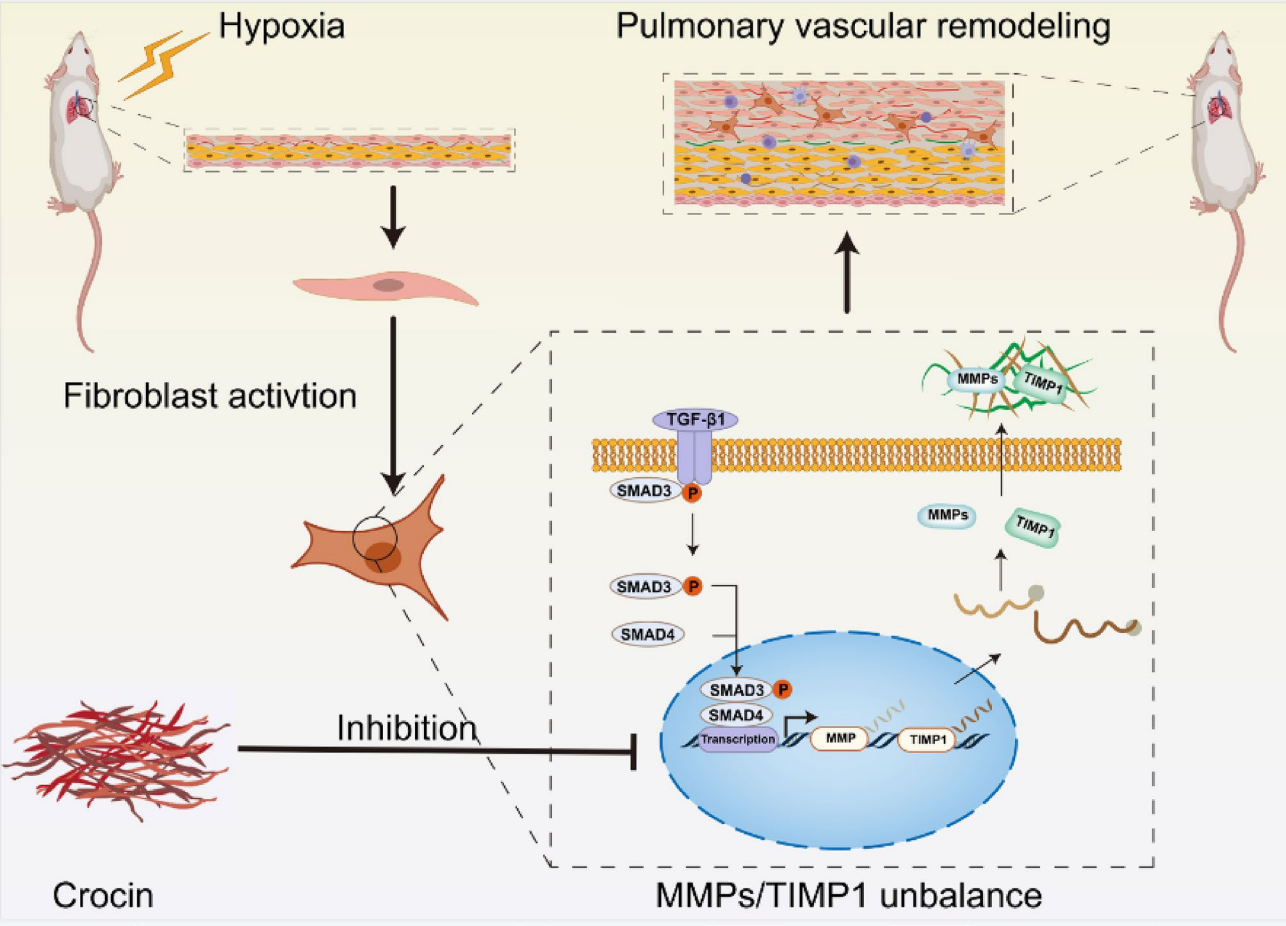
Graphical depiction of crocin attenuating hypoxia-induced pulmonary hypertension via modulating pulmonary arterial fibroblast MMP2/TIMP1 balance.

To explore whether crocin inhibits pulmonary artery fibroblast transformation to myofibroblasts by regulating the TGF-β1/SMAD3 signaling pathway, we performed WB experiments on lung tissues from the hypoxic group administered crocin and found that TGFβ1/SMAD3 signaling was inhibited (Fig. 6a). In addition, we confirmed that crocin inhibits TGFβ1/SMAD3 signaling in vitro. We used PDGF-BB to induce fibroblast activation and then administered crocin; the results were consistent with the in vivo results (Fig. 6b). Moreover, we used TGF-β1 to induce fibroblast activation. TGF-β1 induced pulmonary artery fibroblast transformation to myofibroblasts, and the results showed that *OPN*, *Col1a1*, *Col3a1*, and *MMPs* were upregulated while *Timp1* was downregulated. After cotreatment with crocin, *a-SMA*, *OPN*, *Col1a1*, *Col3a1*, and *MMPs* were downregulated, and *Timp1* was upregulated (Fig. 6c). Furthermore, we applied SRI-011381, an agonist of TGF-β1, before administering TGF-β1 and crocin, and found that SRI partially reversed the effect of crocin on* a-SMA*, *OPN*, *Col1a1*, *Col3a1*, *MMPs*, and *Timp1*. These findings suggest that crocin targets the TGF-β1/SMAD3 signaling pathway, which was also demonstrated by WB (Fig. 6d). Overall, our results indicate that crocin participates in pulmonary arterial fibroblast transformation to myofibroblasts through the TGF-β1/SMAD3 signaling pathway (Fig. 7).

## Discussion

PH is a chronic pulmonary vascular disease characterized by progressively increasing pulmonary arterial pressure, increased vascular resistance, and right ventricular hypertrophy, which eventually leads to right heart failure and death. Over the past two decades, the development of new drugs has driven innovations in PAH therapy. However, most of these therapeutic targets are focused on vasodilation, and treatment for this disease has limitations^31^. Several previous studies have shown that patients treated with these targeted drugs have high mortality rates despite significantly improved short-term outcomes. The aim of this study was to explore the underlying molecular mechanisms involved in PAH and seek novel therapeutic drug targets to improve the quality of existing therapeutic approaches.

Recent transcriptome sequencing and single-cell RNA sequencing (scRNA-seq) studies have been conducted to evaluate complex tissues and cells under biological and pathological conditions to reveal the specific pathogenesis of PAH^32^. Kahori et al. compared 32 mouse strains exposed to chronic hypoxia and identified overlapping DEGs in PL/J, MRL/MpJ, and FVB/NJ mice along with abnormal T-cell expression and increased C5a-C5aR signaling^11^. In addition, Park et al. performed transcriptomic profiling of pulmonary endothelial cells from Sox17-deficient mice and revealed that loss of Sox17 promoted abnormal proliferation and inflammation in lung endothelial cells under hypoxic stress^9^. Rodor et al*.* performed scRNA-seq sequencing of lung endothelial cells isolated from an endothelial lineage tracing mouse model and found that 51% of the DEGs were upregulated in rats or human PAH. Although the above transcriptomic studies and sc-RNA-seq data provide insights into PAH development, there are discrepancies in the identified DEGs and results, potentially due to differences in study design, modeling methods, species, intrasample heterogeneity, and data processing software and algorithms. In the present study, we performed a transcriptome study of C57BL/6 mouse HPH to explore the pathogenesis of PAH and identify key molecules and pathways involved. We applied transcriptome sequencing and GO analysis and found that ECM organization, smooth muscle cell proliferation, oxidative stress, leukocyte migration, and inflammatory response play important roles in PAH development.

The composition of the ECM is regulated by the balance between proteolytic enzymes, such as MMPs, metalloproteinases, serine elastase, lysyl oxidase, and their endogenous inhibitors, TIMPs. In PAH, the imbalance of proteolytic enzymes and their endogenous tissue inhibitors leads to increased collagen deposition, collagen crosslinking, and elastin breakdown in the vascular and perivascular compartments of the pulmonary arteries^33,34^. Benisty et al. reported that the expression of MMPs is significantly increased in the urine of patients with pulmonary hypertension, which may reflect the remodeling of pulmonary vessels^35^. Soban Umar et al. demonstrated that the activation of MMP signaling in a rat model of pulmonary hypertension promoted ventricular hypertrophy and remodeling^36^. In the present study, we constructed a PPI network and screened 10 key genes enriched in DEGs from the lung tissues of HPH mice. Among these genes, TIMP1, a component of the endogenous inhibitor metalloproteinase tissue inhibitor, was downregulated. In addition, the expression of *MMP-2* and *MMP-9* was increased significantly in the mice with HPH, which led to an imbalance in MMPs/TIMP1. Previous studies have shown that this imbalance between MMPs and TIMPs has been proven to induce ECM remodeling in patients with IPAH^37^. In addition, overexpressing adenovirus TIMP1 in MCT-induced pulmonary hypertension in rats reduced pulmonary vascular remodeling, suggesting that balancing MMPs/TIMP1 can reverse the disease^38^. However, another study by the same group in hypoxia-induced pulmonary hypertension in rats found that overexpressing adenovirus TIMP1 aggravated pulmonary hypertension. These contradictory results regarding the TIMP1 under hypoxia and monocrotaline pulmonary hypertension model in rats indicated that the beneficial effect of artificially increasing TIMP1 depended on the primary injury involved and its balance with MMPs. In order to determine whether correcting the MMPs/TIMP1 imbalance can ameliorate pulmonary vascular remodeling, we screened potential natural MMP inhibitors. Previous studies have found that two candidate natural compounds, hesperetin and crocin, can inhibit MMP activity^29^. Among the two candidates, crocin has been reported to attenuate pulmonary inflammation and oxidative stress in a rat model of monocrotaline-induced pulmonary arterial hypertension^24–26^. We confirmed that crocin inhibits pulmonary vascular remodeling and inflammation in HPH, while hesperetin has no protective effect on hypoxia-induced pulmonary hypertension in mice. Regarding the inhibition of hesperetin on pulmonary fibrosis, Li et al. found that hesperetin (200 mg/kg or 400 mg/kg) had a protective effect on silica-induced pulmonary fibrosis; their dose was different from that (50 mg/kg) used in our study. We hypothesize that hesperetin may have protective effects on pulmonary hypertension at a high dose, though further studies with high doses of hesperetin may be needed to confirm this. Additionally, we investigated whether hesperetin affected MMP2/TIMP1 balance in vitro and found that it had no effect on MMP-2/TIMP1 balance (Supplementary Figure 2), which indicates that the mechanism of hesperetin may differ from that of crocin. Previous studies have shown that high doses of crocin proportionally reduce the levels of macrophages and their inflammatory derivatives in atherosclerosis, including MCP-1, TNF-α, IL-6, MMP-2, MMP-3, and MMP-9. In addition, it was found that there was a significant decrease in the MMP-2/TIMP2 ratio after crocin treatment^28^. Soong et al. reported that crocin inhibited fibroblast proliferation; simultaneously decreased α-SMA expression and the mRNA levels of *COL1A1*, *COL3A1*, and *MMP-1*, and increased *Timp1* mRNA levels in bleomycin-induced sclerotic mice, demonstrating the antifibrotic effects of crocin^39^. Combining these findings with those obtained in our study, we hypothesized that crocin can modulate the balance of MMPs/TIMP1 in the pulmonary tissue of HPH mice.

The role of endothelial cells and smooth muscle cells (SMCs) in vascular remodeling has been extensively studied, but relatively little attention has been given to adventitial fibroblasts^40^. Fibroblasts are the main producers of the ECM in all organs and play key roles in the coordination of normal tissue homeostasis and the response to disease^41^. PAF proliferation and differentiation are critical in PAH pathogenesis. Several factors participate in PAF activation. Several studies have shown that the plasma Galectin-3 (Gal-3) level, which is a key fibroblast activation factor, is significantly increased in PAH patients and that Gal-3 expression is upregulated in the adventitia of pulmonary arteries. In addition, inhibition of Gal-3 improved pulmonary vascular remodeling in PAH and simultaneously inhibited the proliferation and differentiation of PAFs^42^. It was also previously shown that inhibiting FABP5 expression in mice abrogates pulmonary artery remodeling and improves heart function in left heart disease-associated pulmonary hypertension, and silencing FABP5 attenuates the TGF-β1-induced fibrosis response in cultured PAFs^43^. Chen et al. reported that 5-HT directly activates PAFs and signals through the TGF-β1/Smad 3 pathway to promote fibroblast activation and adventitial fibrosis, ultimately leading to pulmonary hypertension^41^. Given the above research results, PAF activation likely plays an important role in pulmonary vascular remodeling, and targeting this process may be a new therapeutic approach for treating pulmonary hypertension. Crocin inhibits fibroblast activation and participates in fibrosis in several organs, such as the liver^44^, lung^24,45^, and heart^46^. In the present study, we established a PAF activation model induced by PDGF-BB and clarified whether crocin has an effect on PAF activation. Through BrdU cell proliferation and wound healing cell migration assays, we found that crocin inhibited cell proliferation and migration after administration. Soong et al. reported that crocin inhibited fibroblast proliferation, decreased α-smooth muscle actin (*α-SMA*) expression, reduced the mRNA levels of *COL1A1*, *COL3A1*, and *MMP-1*, and increased the mRNA levels of *Timp1* in bleomycin-induced sclerotic mice, demonstrating the antifibrotic effects of crocin^39^. To further explore whether crocin affects fibroblast activation, we evaluated the expression levels of *α-SMA*, *Col1a1*, *COL3A1*, and *COL5A1* in PAFs. WB revealed that crocin significantly reduced the expression of α-SMA and Col1a1, demonstrating that crocin could inhibit PAF activation.

Dysregulation of TGF-β1 signaling contributes to pulmonary artery remodeling and is thought to promote PAH^33^, particularly by promoting cell proliferation. Dominant-negative mutation of the TGF-β receptor blocks hypoxia-induced pulmonary artery remodeling in mice^47^. Activation of TGF-β1 signaling leads to excessive fibroblast proliferation and infiltration, myofibroblast production, extracellular matrix accumulation, and inhibition of collagen degradation^33,34^. Activation of TGF-β1 induces the phosphorylation of Smad2/3, which forms the Smad complex and interacts with transcription factors, such as α-SMA, to promote gene expression^48–50^. The TGF-β1/SMAD signaling pathway is closely related to cardiovascular diseases^51^. The TGF-β1/SMAD signaling pathway is one of the major inducers of RV fibrosis in MCT-induced pulmonary hypertension^52,53^. The above studies suggest that TGF-β1/SMAD3 signaling is involved in PAH development. In the present study, we demonstrated that crocin inhibits fibroblast activation and extracellular matrix production by inhibiting the activation of the TGF-β signaling pathway. WB analysis of lung tissue revealed that activation of the T GF-β1/SMAD3 signaling pathway was significantly inhibited in the crocin group, and activation of the TGF-β1/SMAD3 signaling pathway was significantly inhibited after the administration of crocin in our fibroblast model. To further verify whether fibroblast activation was associated with crocin, we pretreated cells with an agonist of TGF-β1 and then administered crocin. Compared with that in cells not treated with crocin, the inhibitory effect of crocin was partially restored when the agonist was used. Taken together, these findings indicate that crocin modulates TGF-β1/SMAD3 signaling in PAFs, which is the molecular mechanism through which crocin regulates MMP2/TIMP1 homeostasis to inhibit pulmonary vascular remodeling.

Our study has some limitations. We demonstrated the potential protective effect of crocin; however, we did not determine whether crocin can reverse established HPH. In addition, we only applied one dose of crocin in the present study. According to the previously published literature, further studies are needed to determine the optimal dose and ideal therapeutic course of crocin.

Given our results, we conclude that crocin can prevent HPH development in hypoxic mice. We presented new data showing that crocin attenuates pulmonary hypertension, pulmonary vascular remodeling, and RV hypertrophy in HPH mice, likely through blockade of hypoxia-induced hyperactivity of TGF-β1/Smad3 signaling and inhibition of fibroblast activation. This provides a potential therapeutic method for the treatment of pulmonary hypertension in people with chronic hypoxia-related diseases (such as obstructive pulmonary disease, bronchiectasis, altitude sickness, and sleep-related respiratory disorders).

## Materials and methods

### Hypoxia-induced pulmonary hypertension in mice

Male 6–8-week-old C57BL/6 mice were purchased from HFK Bioscience Company (Beijing, China), and hypoxia-induced pulmonary hypertension was induced as described previously^54^. Hesperetin and crocin (Biopurify Phytochemical, Chengdu, China) were intraperitoneally injected at a dose of 50 mg/kg body weight every 3 days. After 4 weeks, the right ventricular systolic pressure (RVSP) was measured, and the right ventricular hypertrophy index was calculated [RVHI = RV/(LV + S) × 100%]. The study was conducted in accordance with the ARRIVE guidelines. The animal experiments in this study were approved by the Beijing Anzhen Hospital Ethics Committee, and the experimental procedures were conducted in accordance with the National Institutes of Health Guide for Care and Use of Laboratory Animals.

### Cell culture

Mouse pulmonary arterial fibroblasts (Procell, Wuhan, China) were maintained in fibroblast medium (ScienCell, San Diego, CA, USA), and SL4 mouse colon cancer cells were maintained in Dulbecco’s modified Eagle medium (DMEM)/F12 (Gibco, New York, USA) supplemented with 10% fetal bovine serum (FBS, Gibco, New York, USA) and 1% penicillin and streptomycin (Gibco, NY, USA) as described previously^55^. All cells were maintained at 37 °C in 95% humidified air and 5% CO2. Recombinant TGF-β1 (Peprotech, NJ, USA) was used at a dose of 5 ng/ml, recombinant PDGF-BB (MCE, NJ, USA) was used at a dose of 20 ng/ml, and SRI-011381 (MCE, NJ, USA) was used at a dose of 10 μM to treat PAFs.

### BrdU assay

Fibroblasts were pretreated with 10 or 50 μM crocin, and then stimulated with PDGF-BB. Then, BrdU (10 μM) was added within 2–4 h before the end of treatment. After 24 h of drug administration, the cells were fixed and incubated with primary BrdU antibody (Zhongshan Golden Bridge, Beijing, China; 1:200) at 4 °C overnight. Afterward, the cells were incubated with a FITC-labeled secondary antibody (Invitrogen, CA, USA; 1:1000) for 1 h at room temperature. The nuclei were stained with DAPI and detected and analyzed with an ImageXpress XK Microscale (Molecular Devices, CA, USA).

### Cell scratch migration assay

Fibroblasts were seeded and scratched with a 200 μL sterile pipette tip 24 h later. The cells were treated with 10 or 50 μM crocin for 30 min, and then stimulated with 20 ng/ml PDGF-BB. The wound area was observed with an inverted light microscope (Leica, Wetzlar, Germany) at 0, 24, and 48 h and was analyzed using ImageJ software (National Institute of Health, MD, USA).

### Western blotting (WB)

Lung tissue and cell samples were prepared, and WB was performed as previously described^56^. The primary antibodies used were as follows: MMP9 (1:1000; Biorbyt, Britain), MMP2 (1:1000; CST, USA), TIMP1 (1:1000; Abcam, USA), MMP3 (1:1000; CST, MA, USA), TGF-β1 (1:1000; Abcam, USA), P-smad3 (1:1000; CST, MA, USA), T-smad3 (1:1000; CST, MA, USA), P-smad2 (1:1000; CST, MA, USA), and T-smad2 (1:1000; CST, MA, USA).

### Real-time PCR

Total RNA was extracted from lung tissue and cells using FreZOL reagent (Vazyme, Nanjing, China). cDNA generation and q-PCR analyses were performed using SYBR Greener qPCR SuperMix Universal (Invitrogen, Carlsbad, CA, USA) according to the manufacturer’s instructions. The relative quantification of gene expression (for MMP2, MMP3, MMP9, TIMP1, SPP1, Col1a1, Col3a1, Col5a1, and A-SMA) was determined by comparison with the relative endogenous reference gene GAPDH. The specific primer set sequences are listed in Table 2.

**Table 2:** The sequences of the mouse primers used for real-time PCR.

Gene	Forward	Reverse
α-SMA	5′-CTGACAGAGGCACCACTGAA-3′	5′-CATCTCCAGAGTCCAGCACA-3′
SPP1	5′-AGCAAGAAACTCTTCCAAGCAA-3′	5′-GTGAGATTCGTCAGATTCATCCG-3′
Col1a1	5′-GAGCGGAGAGTACTGGATCG-3′	5′-GTTCGGGCTGATGTACCAGT-3′
Col3a1	5′-CCCCTGGTTCTTCTGGACAT-3′	5′-TGGGCCTTTGATACCTGGAG-3′
Col5a1	5′-AGATGGCATCCGAGGTCTGAAG-3	5′-GACCTTCAGGACCATCTTCTCC-3′
TIMP1	5′-CGAGACCACCTTATACCAGCG-3′	5′-ATGACTGGGGTGTAGGCGTA-3′
MMP-2	5′-CGATGTCGCCCCTAAAACAG-3′	5′-GCATGGTCTCGATGGTGTTC-3′
MMP-3	5′-GTTCTGGGCTATACGAGGGC-3′	5′-TTCTTCACGGTTGCAGGGAG-3′
MMP-8	5′-ACCAGTGCTGGAGATATGACA-3′	5′-ACTCCTGGGAACATGCTTGG-3′
MMP-9	5′-TGGGCGTTAGGGACAGAAAT-3′	5′-GAACCATAACGCACAGACCC-3′
GAPDH	5′-AATGCATCCTGCACCACC-3′	5′-ATGCCAGTGAGCTTCCCG-3′

### Gelatin zymography assay

MMP activity was measured using a Gelatinase/Collagenase assay kit (Real-Times, Beijing, China). The cell supernatant was treated and electrophoresed on an 8% sodium dodecyl sulfate‒polyacrylamide gel with 0.1% gelatin. Then, the gel was treated and stained with Coomassie blue R-250 solution as previously described^29^.

### Histopathology

The lung tissues were fixed, embedded, and sectioned as previously described^56^ and stained with an HE staining kit (Zhongshan Gold Bridge, China). The distal pulmonary artery (with a diameter of 50–150 μm) wall thickness ratio (distal pulmonary artery wall thickness ratio, MWT%) was calculated as follows: MWT% = [(outer pipe diameter)/outer pipe diameter] 100%. The sectioned lung tissues were subjected to Masson staining (Solebo, Beijing, China) or immunohistochemistry (IHC) with antibodies against TIMP1 (Abcam, Cambridge, UK) and MAC-3 (Santa Cruz Biotechnology, Dallas, TX, USA) as previously described^56^. For immunofluorescence staining, mouse lung tissues were incubated with antibodies against MMP2, TIMP1, and FSP (Abcam, Cambridge, UK) at 4 °C overnight. Subsequently, FITC- or TRITC-conjugated secondary antibodies (Jackson ImmunoResearch, West Grove, PA, USA) were applied at room temperature for 1 h. Images were obtained with a confocal fluorescence microscope (Leica Microsystems, Buffalo Grove, IL, USA).

### Transcriptome analysis

Lung tissue RNA was extracted from the hypoxic group (n = 4) and normoxic group (n = 3) using TRIzol (Thermo Fisher Scientific, Waltham, MA, USA) according to the manufacturer’s instructions. Whole-transcriptome sequencing was completed by BGI Genomics Co., Ltd. (Wuhan, China) using the BGISEQ-500 platform. The data were filtered with the filtering software SOAPnuke (version 1.5.2) developed by BGI Genomics.

### Protein‒protein interaction (PPI) network analysis and identification of hub genes

The STRING protein database (https://www.string-db.org/) was used for online analysis of the PPI network. Protein interaction data were analyzed with Cytoscape (version 3.8.0) software, the CytoHubba plug-in was used to construct the PPI subnetwork, and the top 10 genes were screened as hub genes according to the topological analysis method of connectivity (degree).

### Statistical analysis

Continuous data are presented as the mean ± standard deviation, and a *t* test was used for the comparison of two independent groups. P < 0.05 was considered to indicate significance. GraphPad Prism 7.0 was used for statistical analyses of the data. The R language and corresponding R software packages were used for bioinformatic analysis and visualization.

### Supplementary Information

Supplementary Figures.

Supplementary Legends.

## Data Availability

The raw sequence data reported in this paper have been deposited in the Genome Sequence Archive (Genomics, Proteomics & Bioinformatics 2021) at the National Genomics Data Center (Nucleic Acids Res 2022) and the China National Center for Bioinformation/Beijing Institute of Genomics, Chinese Academy of Sciences (GSA: CRA015863). They are publicly accessible at https://ngdc.cncb.ac.cn/gsa.

## Abbreviations

CRO: Crocin
HPH: Hypoxic pulmonary hypertension
TIMP1: Tissue inhibitor of metalloproteinase
MMPs: Matrix metallopeptidases
PDGF-BB: Platelet-derived growth factor-BB
TGF-β1: Transforming growth factor-β1
SMAD: Small mothers against decapentaplegic
