# Supplementary figures and images for: Crocin's role in modulating MMP2/TIMP1 and mitigating hypoxia-induced pulmonary hypertension in mice
Source: Sci Rep. 2024 Jun 3;14:12716. doi: 10.1038/s41598-024-62900-8 (PMC11148111; doi:10.1038/s41598-024-62900-8)

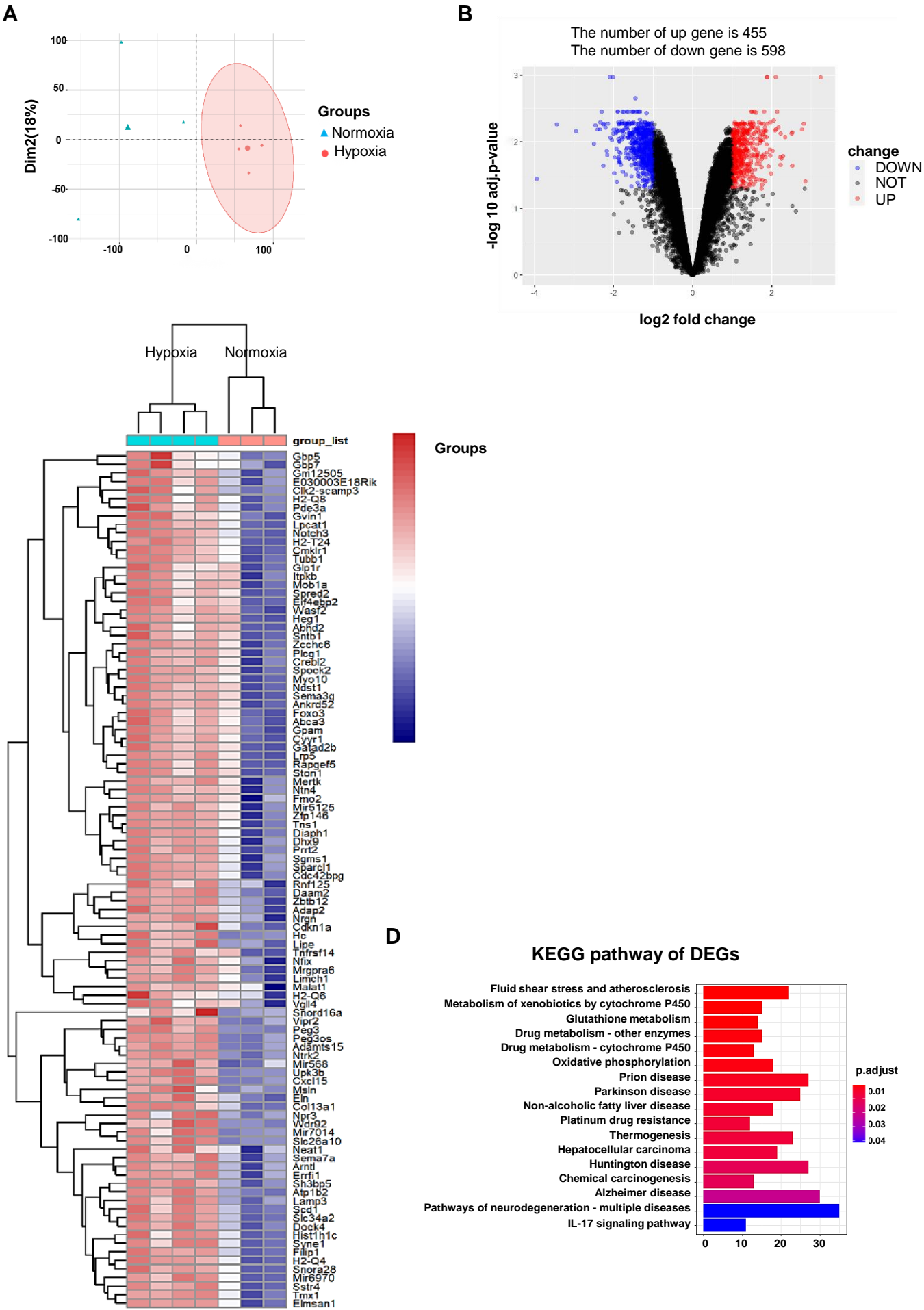

Supplementary Fig 1

A

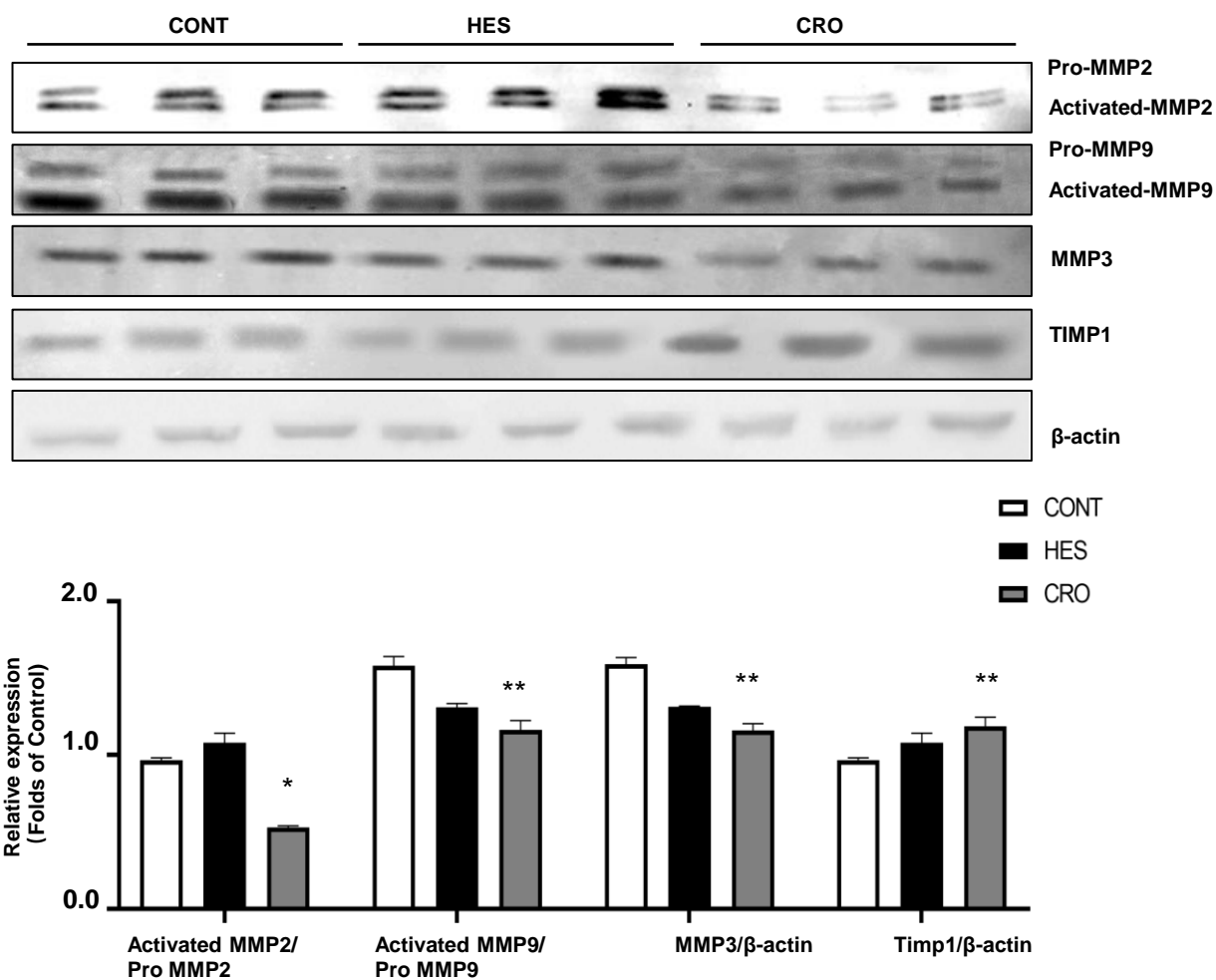

B

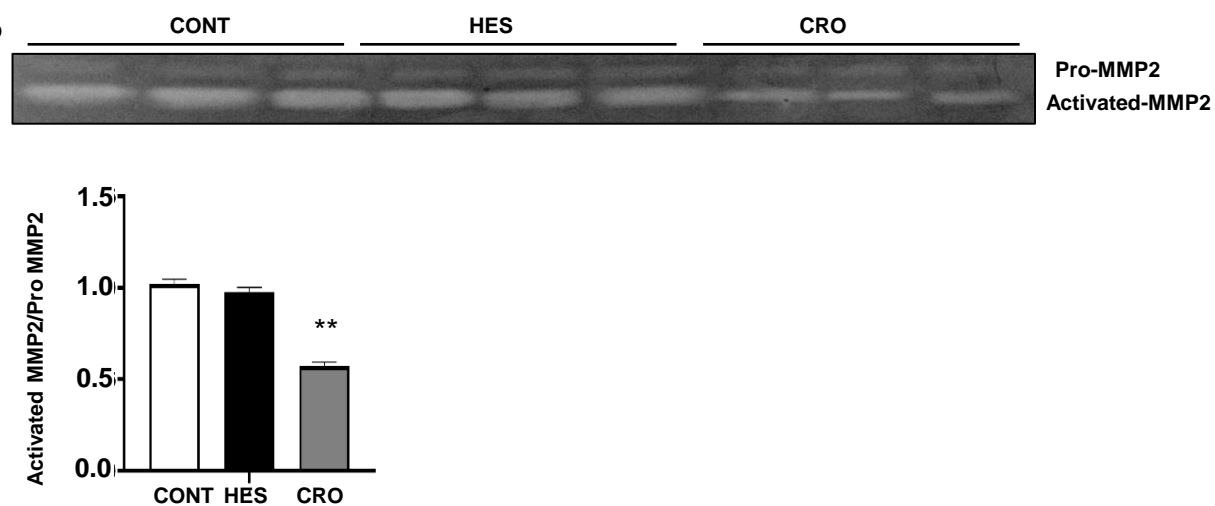

Supplement: Supplementary file 1 — Supplementary Figures. [file 41598_2024_62900_MOESM1_ESM.pdf]
