# Supplementary material for: Crocin's role in modulating MMP2/TIMP1 and mitigating hypoxia-induced pulmonary hypertension in mice
Source: Sci Rep. 2024 Jun 3;14:12716. doi: 10.1038/s41598-024-62900-8 (PMC11148111; doi:10.1038/s41598-024-62900-8)
Supplement: Supplementary file 2 — Supplementary Legends. [file 41598_2024_62900_MOESM2_ESM.docx]

**Supplementary Figure legends**

**Supplementary Figure 1. Trancriptomic analysis of lung tissue from mice with hypoxia-induced pulmonary hypertension**

(A) principal component analysis (PCA) of each sample of lung tissue from the hypoxia group (n = 4) and normoxia groups (n = 3). (B) Differentially expressed gene volcano plot with each point in the coordinate system representing a gene. The abscissa represents the fold change of gene expression in the mice from the hypoxia group after considering the log2 logarithm (compared with the mice in the normoxia group), whereas the ordinate represents the difference analysis. The corrected p-values are −log10 log-transformed values, with red dots indicating upregulated genes and blue dots indicating downregulated genes. (C) Heatmap of the top 100 differentially expressed genes clustering, with the horizontal axis representing each sequenced sample, the vertical axis representing the gene, red representing upregulated expression of the gene in pulmonary hypertension, and blue representing downregulation, the darker the color, the greater the fold change of upregulation. (D) KEGG pathway enrichment analysis results for differentially expressed genes.

**Supplementary Figure 2. Crocin regulates MMP/TIMP1 homeostasis in mice colorectal cancer SL4 cell line.**

(A) The effects of crocin on MMP and TIMP1 homeostasis in mice colorectal cancer SL4 cell line, crocin or hesperetin (50 μM) compared with control group; ** P < 0.01; * P < 0.05. (B) Effect of crocin on MMP-2 activity in mice colorectal cancer SL4 cell line administered crocin or hesperetin (50 mg/kg) compared with that in the control group; ** P < 0.01.
